# Effects of mindfulness and fatigue on emotional processing: an event-related potentials study

**DOI:** 10.3389/fnbeh.2023.1175067

**Published:** 2023-05-25

**Authors:** Jialin Fan, Wenjing Li, Mingping Lin, Xinqi Li, Xinmei Deng

**Affiliations:** ^1^School of Psychology, Shenzhen University, Shenzhen, China; ^2^The Shenzhen Humanities and Social Sciences Key Research Bases of the Center for Mental Health, Shenzhen, China

**Keywords:** mindfulness, fatigue, emotion, emotion processing, event-related potentials

## Abstract

Fatigue is a common experience in everyday life. People who experience fatigue will have more intense negative emotions, and at the same time, their positive emotions will decrease, impairing the individual’s emotional processing ability. In previous research, mindfulness meditation reduces the intensity of negative emotional stimuli. However, if individuals continue to be affected by negative emotions when they are fatigued, it is unclear whether mindfulness can buffer the negative association between fatigue and emotions. This study examined whether mindfulness meditation affects the association between fatigue and emotions, using event-related potentials (ERPs). One hundred and forty-five participants completed the experiment. They were randomly assigned to the Mindfulness or Non-mindfulness group; and they were presented with positive, neutral, or negative pictures in an emotional processing task before and after mindfulness or rest. Late positive potential (LPP) is an important indicator of emotional stimuli perceived by individuals, and positive or negative pictures can induce an increase in LPP amplitude more than neutral pictures. Our findings suggest that fatigue significantly affected individuals’ LPP amplitudes in the early, mid, and late windows in the Non-mindfulness group, specifically, the more fatigued individuals had lower LPP amplitudes, but not in the Mindfulness group. These results suggest that in a state of fatigue, mindful individuals are able to maintain responsiveness to emotional stimuli by maintaining LPP amplitude. Our study has demonstrated that mindfulness meditation, to some extent, offsets the negative association of fatigue with the neural activation of emotions.

## 1. Introduction

Occupations such as counselors, human resources advisers, customer service staff, and nurses often suffer from fatigue due to the high emotional demands of their work tasks (also called emotional workload). They have a high level of exposure to negative emotions from clients, customers, or members of the public who seek assistance and support. Their emotions affect their job performance and efficiency, the quality of services they provide, and the satisfaction of their customers, and they also have a critical impact on their mental health. Mindfulness, an attentive, non-judgmental focus on present experiences, is increasingly used in the workplace (Bartlett et al., 2019) to help staff foster emotional regulation. However, there is little relevant literature on the relationship between fatigue, mindfulness, and emotional processing. It is unclear whether mindfulness still has a role in emotional processing in individuals in a fatigued state, or whether fatigue moderates the effect of mindfulness on emotion.

Fatigue is a common phenomenon experienced in our daily lives, but it is also a complex state involving changes in motivation and emotion, behavior, and information processing (van der Linden, 2011). Although there is no unified definition of fatigue from the last century to the present, there is some consensus that, in terms of consequences, individuals are subjectively symptomatic, feel uncomfortable or fatigued, and are reluctant to engage in further activities, with symptoms including slower reactions, reduced judgment, and deteriorating attention (e.g., Cercarelli and Ryan, 1996; Beurskens, 2000; Enoka and Duchateau, 2016; Fan and Smith, 2020).

At the level of explicit behavior, there is some association between fatigue and emotional processing. Research has shown that the emotional responses of individuals experiencing fatigue can range from impatience to emotional numbness (Olson, 2007). And in cases of chronic fatigue, individuals are more likely to repress or hide their emotions, resulting in a reluctance to seek social support (Chalder and Hill, 2012; Brooks et al., 2017). Furthermore, individuals who experienced mental fatigue after the prolonged cognitive activity had significantly more fatigue and negative emotions and significantly less attention and positive emotions during the task (Li et al., 2016).

The emotional response is a complex process, its physiological manifestations can be measured by event-related potentials (ERPs). High temporal resolution ERP captures rapid neural responses and, through objective physiological indicators, helps researchers better understand emotions and their processes. The visually evoked late positive potential (LPP) is one of the ERP components modulated by the emotional intensity of the stimulus and serves as one of the neurophysiological indicators of emotional processing. The LPP appears approximately 300 ms after the stimulus, and studies have noted that the early time window of the LPP (300–1,000 ms) is primarily concerned with attention allocation, and the late time window (>1,000 ms) indicates the memory and meaning formation stages (Moser et al., 2014). In the cognitive domain, early findings of great significance suggest that increases in LPP amplitude are related to the meaning of task-relevant stimuli (Johnson, 1988; Picton, 1992). The LPP is a strong sign of conscious recognition because it is sensitive to the cognitive appraisal of stimulus meaning and the allocation of attentional resources (Luck and Hillyard, 2000; Kranczioch et al., 2003; Yuan et al., 2007; Zhang et al., 2015). From an incentive attention perspective (Lang et al., 1997), emotional cues automatically select and utilize attentional processing resources when eliciting an individual’s emotional response (Cuthbert et al., 2000; Keil et al., 2002). On the other hand, many studies have shown that changes in LPP amplitude reflect the degree of arousal of the emotional stimulus itself and the increase in individual attention to the emotional stimulus during emotion processing (Lang et al., 1997; Schupp et al., 2006; Wessing et al., 2013). Specifically, it has been found that when positive and negative pictures are presented to individuals, their amplitude is greater than when neutral pictures are presented (Cuthbert et al., 2000; Schupp et al., 2003, 2004). Therefore, we can assume that the effect of the response to an emotional stimulus increases the amplitude of an individual’s LPP and is a sign that the individual perceives the emotional stimulus and reflects its intrinsic meaning.

However, at the neuroscientific level, there are few studies linking fatigue to emotion, and only a few studies have shown that to some extent a decrease in LPP is associated with fatigue. Van Dillen and Derks (2012) found that individuals with a high working memory load exhibited reduced LPP amplitude in response to emotional faces. P300 and N2 are ERPs, the specific evoked potential that is associated with cognitive function. P300 is a positive wave that appears approximately 300 ms after the event (e.g., auditory and visual stimuli), and N2 appears before P3; and is the negative wave that appears about 200 ms after the event, reflecting the brain’s initial processing of stimuli. N2 and P3 are not influenced by the physical properties of the stimuli and are related to the mental state and attention of the participants. Garrison et al. (2017) found that individuals who completed a complex writing task showed more negative N2 amplitude, and attention span on visual stimuli reduced after a brief (5-min) period of mental exertion. This finding suggests that lowering P3 or LPP may require a longer and more taxing mental effort.

Mindfulness meditation is a strategy to deal with emotions and has been increasingly used to improve the regulation of emotions and attention in the workplace. Mindfulness is being consciously aware of one’s experience of the present moment here and now and accepting all one’s feelings without judgment of that experience (Kabat-Zinn, 2003). Bishop et al. (2004) suggested that mindfulness functions in two important ways:

1. The control of attention. The control of one’s attention to keep it in the present moment, including the awareness of one’s emotions, behavior, and consciousness, and also the control of one’s awareness to keep it properly focused.
2. The orientation to the experience is the open and accepting attitude toward the perceived experience.

In the workplace, major corporations, such as Google and Apple, may have been among the first to offer mindfulness meditation programs to their employees, with a number of other Fortune 500 corporations having followed. Dane and Brummel (2014) conducted a study in the workplace; and found a positive relationship between workplace mindfulness and job performance. Researchers (Lomas et al., 2017) systematically reviewed 153 of the empirical literature on work settings and found that mindfulness was generally associated with positive outcomes on most measures, although the number of high-quality studies was limited. Nevertheless, King (2019) criticized that, the growing trend in major corporations to utilize such workplace programs, and the growth in the number of for-profit firms offering such programs may inevitably lead to exaggerated claims about the benefits of mindfulness.

Mindfulness is beneficial to emotional processing. Previous studies have demonstrated that individuals trained in mindfulness meditation have lower LPP amplitudes in response to negative stimuli. It suggests that mindfulness meditation protects the individual from strong damage in the face of negative emotional stimuli, and promotes the health of individual emotional functioning (Sobolewski et al., 2011; Brown et al., 2013; Lin et al., 2016; Deng et al., 2020). Mindfulness has also been found to facilitate emotional processing during attention and regulation (Goldin and Gross, 2010; Schoenberg, 2016; Kaunhoven and Dorjee, 2017). Individuals who engage in long-term mindfulness meditation were found to have their cognitive abilities (Cahn and Polich, 2006) and emotions (Davidson et al., 2003) improved, while short-term mindfulness meditation can also promote attentional stability and enhance the negative moods of individuals (Zeidan et al., 2010). Subsequent studies have revealed that mindfulness meditation practice improves the emotional experience by enabling attentional processing, increasing the ability to maintain attention in the present moment (Reva et al., 2014), focusing on physical and emotional experiences, and producing a shift in thinking (Majumdar et al., 2002; Shapiro et al., 2006; Pavlov et al., 2015).

Mindfulness also works on fatigue. Previous studies suggested that mindfulness meditation can partially replace rest or sleep and alleviate mental fatigue’s adverse effects (Zeidan et al., 2010; Thomas, 2019; Kudesia et al., 2022). However, it remains unclear whether mindfulness has a buffering to the negative association with fatigue and emotions. In a real-life setting, employees may fatigue during or after highly emotional demands of work, but still, have to maintain high emotional responsiveness with clients. In these cases, would mindfulness meditation be able to improve fatigued employees’ emotions?

In the present study, we brought together the converging lines of evidence in mindfulness, fatigue, and emotions by using ERP. This study aimed to understand how mindfulness meditation affects the negative association between fatigue and emotions. We, therefore, examined the neural responses of participants with mindfulness meditation and participants without mindfulness meditation in an emotional processing task. Associations between fatigue and the neural responses to different emotional stimuli were compared between the Mindfulness and the Non-mindfulness groups. According to the prior literature (Van Dillen and Derks, 2012; Garrison et al., 2017), the reduction of the negative correlation between fatigue and individuals’ emotions is achieved by reducing neural activation and response to emotional stimuli. Mindfulness meditation can weaken the negative influence of mental fatigue (Zeidan et al., 2010; Thomas, 2019; Kudesia et al., 2022). In this case, we hypothesized that there were negative associations between fatigue and the neural responses to emotional stimuli during emotional processing in the Non-mindfulness group. Negative correlations between participants’ level of fatigue and the amplitudes of LPPs in the emotional processing task were expected. In terms of the buffering function of mindfulness to the negative association between fatigue and emotions, we expected no significant correlation between participants’ level of fatigue and the amplitudes of LPPs.

## 2. Materials and methods

### 2.1. Participants

A total of 152 Chinese undergraduates were recruited to participate in studying emotions and mindfulness. The sample size was close to the optimal sample size calculated in G*Power (Faul et al., 2007) for ANCOVA, *f* = 0.25, *a* = 0.05, 1-beta = 0.80), resulting in *N* = 158. Participants were randomly assigned to either a Mindfulness group or a Non-mindfulness group. Seven of the participants were excluded from the analysis due to excessive artifacts in the EEG recordings. The final sample thus consisted of 145 participants, including 74 in the Mindfulness group (48 females and 26 males, aged from 18 to 24 years old, *M*_*age*_ = 20.43, *SD* = 1.60) and 71 in the Non-mindfulness group (47 females and 24 males, aged from 17 to 25 years old, *M*_*age*_ = 20.27, *SD* = 1.69).

**FIGURE 1:**
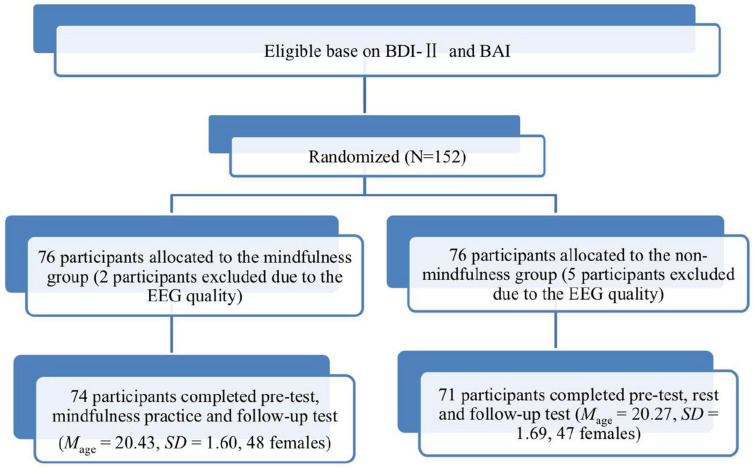
Shows the flowchart of participants in the study.

All participants were healthy, right-handed, and had normal or corrected-to-normal vision. The participants had no history of neurological or psychiatric disorders and had not engaged in mindfulness practice. The participants had normal anxiety or depression scores as indicated by the low scores (BDI-II < 10, MBDI-II = 5.62; BAI score < 45, MBAI = 18.04) in the measures of the Beck Anxiety Scale (BAI; Beck et al., 1988) and the Beck Depression Inventory-II (BDI-II; Beck et al., 1996). The research protocol of the present study was approved by the Shenzhen University Institutional Review Board (IRB) prior to data collection (PN-202200014). Informed consent was obtained before the study began. Figure 1 shows the flowchart of the participants of the study.

### 2.2. Stimuli

One hundred thirty-five emotional pictures from the Chinese Affective Picture System (CAPS) (Bai et al., 2005) were selected as stimuli, including 45 positive (e.g., happy face), 45 negative (e.g., fierce snake), and 45 neutral (e.g., a broom) non-repeating pictures. The valence and arousal of all stimulus pictures were rated on a scale of 1–9 by 30 experts in the field of emotion. The results showed significant differences in the valence dimension among the three categories (*p* < 0.001; *M* ± *SD*: Positive = 7.20 ± 0.31; Negative = 2.53 ± 0.39; Neutral = 5.48 ± 0.26). The arousal ratings in response to positive and negative pictures were significantly higher than that of neutral pictures (both *p* < 0.001; *M* ± *SD*: Positive = 5.70 ± 0.53; Negative = 5.51 ± 0.61; Neutral = 3.71 ± 0.48). The level of arousal ratings in response to positive and negative pictures was not significant (*p* = 0.059). The pictures (330 × 340 pixels) were presented in color by a 21-in monitor that occupies approximately 35° of the visual angle horizontally and vertically using E-Prime software.

### 2.3. Affective picture processing task

**FIGURE 2:**
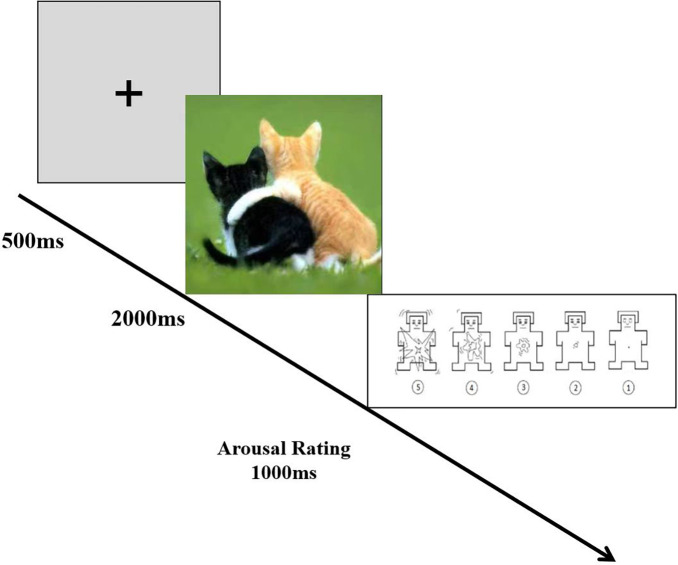
Sample stimuli and procedure.

The affective picture-processing task (Dennis and Hajcak, 2009) effectively assessed participants’ neural responses to different emotional stimuli. As shown in Figure 2, at the beginning of each trial, a fixation point (+) was presented for 500 ms, followed by a target picture for 2,000 ms. The participants were then asked to rate the level of arousal in response to each image (on a scale of 1–5) using a self-assessment manikin (SAM) (Lang et al., 1999). The arousal rating was based on the strength of their feelings (e.g., how strongly did you feel after viewing the picture?).

A total of 135 images were displayed, including 45 positive, 45 negative, and 45 neutral. Before the formal experiment, a practice procedure was used to ensure that participants were familiar with the task. Pictures that appeared in the practice procedure were not included in the formal experiment. The formal test consisted of three blocks, each consisting of 45 trials. Each session required 30–35 min.

### 2.4. Procedures

We employed a 2 × 2 × 3 mixed design: (Group: Mindfulness group vs. Non-mindfulness group) × 2 (Time: before mindfulness meditation or rest vs. after mindfulness meditation or rest) × 3 (Valence: positive vs. negative vs. neutral). Group was the within-subjects factor, and Time and Valence were the within-subject factors. After completing the demographic information, we assessed participants’ fatigue (on a scale of 0–9) using a single question (“How exhausted are you feeling right now?”). Next, electroencephalograph (EEG) sensors were attached to the participants, who were asked to complete the picture-processing task. Then, participants were randomly assigned to either the Mindfulness group or the Non-mindfulness group. Participants in the Mindfulness group completed a 15-min mindfulness meditation. Participants in the Non-mindfulness group took a 15-min rest with their eyes closed. After mindfulness meditation or rest, the participants finished the affective picture-processing task again.

### 2.5. Fatigue measurement

The measurement of fatigue is a daunting task for researchers due to the complex definition of fatigue. In our study, we used a single-item subjective measure that allowed participants to subjectively assess their level of fatigue. For example, how tired do you feel right now? (on a scale of 0–9). In the past, researchers have argued that subjective measures of fatigue cannot truly measure an individual’s fatigue (Cameron, 1973).

However, we can learn from the definitions related to fatigue that fatigue is closely related to the subjective feelings of individuals. Also, according to Aaronson et al. (1999), the salient features of fatigue that need to be considered when measuring fatigue are subjective effects, such as subjective quantification of fatigue, subjective distress caused by fatigue, and subjective assessment of the impact of fatigue on activities of daily living. Furthermore, Youngblut and Casper (1993) provided very strong evidence for the validity and reliability of single-item measurement studies. Many subsequent studies have also shown that the use of single-item subjective measures of individual fatigue has good reliability and validity and contributes to the understanding of individual subjective distress and individual fatigue (Van Hooff et al., 2007; Fan and Smith, 2017; Kim and Abraham, 2017). In addition, we did not use some objective experimental paradigms to measure participants’ fatigue, such as psychomotor vigilance task paradigms, reaction time tests, cognitive tests, or psychophysiological methods that typically use blink rate, heart rate, and breathing in the present study. This is because the use of experimental paradigms or psychophysiological methods to measure individual fatigue may be influenced by the individual’s emotion, respiration, etc., and also increase the experimental burden on the participants.

### 2.6. Mindfulness meditation

Mindfulness meditation was based on and modified from earlier research (Deng et al., 2019). The mindfulness meditation was conducted with audio guidance recorded with a soothing voice by senior mindfulness instructors. It involved a breath-based mindfulness exercise and a body exploration exercise. In the breath-based mindfulness exercise, participants try to pay attention to the sensations that accompany their breathing, to their nostrils, or the movement of the abdomen, without any control of breathing. In the body scan exercise, participants move their consciousness from one part of the body to another, allowing them to experience their body sensations in-depth without judgment. The full version of the audio guide for mindfulness meditation is presented in Supplementary Material 1.

### 2.7. Psychophysiological recording, data reduction, and analysis

A 32-channel amplifier (BrainAmp, Brain Products, Germany) based on the 10/20 system was used to record continuous EEG data with two electrodes placed on the left and right mastoids (TP9 and TP10). The EEG was sampled at 500 Hz, and the impedance was kept below 5 kΩ. The data were re-referenced offline to the averaged mastoid references. The bandpass was filtered from 1 to 40 Hz. Ocular movements and blinking artifacts were corrected using the Independent Component Analysis (ICA) algorithm implemented in Brain Vision Analyser 2.0 (“Brain Products,” Germany). The EEG data from each trial was segmented in epochs from 500 ms before stimuli onset until 2,000 ms after the onset. Trials with artifacts exceeding ±80 μV were excluded from further analysis. The ERPs were averaged separately, based on the different experimental conditions.

Based on the existing literature, LPP was defined as the average activity at Pz (Murata et al., 2013) in three-time windows (O’Hare et al., 2017) after stimulus onset: LPP 300–600 ms (early window), LPP 600–1,000 ms (middle window), and LPP 1,000–1,500 ms (late window).

To examine the effect of mindfulness on the arousal rating at the behavioral level, arousal ratings of emotional experience under the different experimental conditions between different groups were examined using a 2 (Group: Mindfulness group vs. Non-mindfulness group) × 2 (Time: before mindfulness meditation or rest vs. after mindfulness meditation or rest) × 3 (Valence: positive vs. negative vs. neutral) repeated measures ANOVA. For the neural data, repeated ANOVA was performed with Valence (positive vs. negative vs. neutral) and Time (before mindfulness meditation or rest vs. after mindfulness meditation or rest) as the within-subject variable, group (Mindfulness group vs. Non-mindfulness group) as the between-subject variable, fatigue as the covariable, and LPP amplitude as the dependent variable. The IBM Statistical Package for the Social Sciences (SPSS) 20.0 was used to analyze all the data statistically. The degree of significance *p* < 0.05 was chosen. During the analysis, the Bonferroni test was used for multiple *post hoc* comparisons. A Greenhouse–Geisser correction was applied to the *p*-values associated with multiple comparisons.

## 3. Results

### 3.1. Behavioral results

Table 1 shows the average arousal of participants in the Mindfulness and Non-mindfulness groups under different conditions.

**TABLE 1:** Arousal of the mindfulness and Non-mindfulness groups under different conditions.

Time	Non-mindfulness group (*M* ± *SD*)			Mindfulness group (*M* ± *SD*)		
	**Positive**	**Negative**	**Neutral**	**Positive**	**Negative**	**Neutral**
Before mindfulness meditation or rest	2.66 ± 0.69	3.50 ± 0.71	1.65 ± 0.43	2.47 ± 0.71	3.22 ± 0.81	1.59 ± 0.48
After mindfulness meditation or rest	2.43 ± 0.73	3.12 ± 0.87	1.48 ± 0.44	2.25 ± 0.73	2.91 ± 0.90	1.44 ± 0.48

We employed a 2 × 2 × 3 repeated measures analysis of variance (ANOVA) to analyze the average arousal: 2 (Group: Mindfulness group vs. Non-mindfulness group) × 2 (Time: before mindfulness meditation or rest vs. after mindfulness meditation or rest) × 3 (Valence: positive vs. negative vs. neutral). The primary effect of *Valence* was significant, *F*(2,286) = 501.74, *p* < 0.001, η_*p*_^2^ = 0.78. The arousal ratings of the negative stimuli were higher than the positive and neutral stimuli (both *p* < 0.001). The arousal ratings of the positive stimuli were higher than neutral stimuli (*p* < 0.001). The main effect of *Time* was significant, *F*(1,143) = 91.95, *p* < 0.001, η_*p*_^2^ = 0.39. After mindfulness meditation or rest, the arousal ratings were significantly lower than before mindfulness meditation or rest (*p* < 0.001). The effect of the *Group* was not significant, *F*(1,143) = 3.04, *p* = 0.084, η_*p*_^2^ = 0.02.

The interaction between *Valence* and *Time* was significant, *F*(2,286) = 17.84, *p* < 0.001, η_*p*_^2^ = 0.11. *Post hoc* analysis showed that both the Mindfulness and Non-mindfulness groups significantly dropped on the arousal of all valences from the pre-test to post-test (*p*s < 0.001). For all valences, the arousal ratings of the negative stimuli were higher than the positive and neutral stimuli (both *p* < 0.001), and the arousal ratings of the positive stimuli were higher than neutral stimuli (*p* < 0.001) in both the Mindfulness and Non-mindfulness groups. The interaction between *Time* and *Group* was not significant, *F*(1,143) = 0.46, *p* = 0.499, η_*p*_^2^ = 0.00. The interaction between *Valence* and *Group* was not significant, *F*(2,286) = 1.80, *p* = 0.167, η_*p*_^2^ = 0.01. The interaction between *Valence*, *Time*, and *Group* was not significant, *F*(2,286) = 0.46, *p* = 0.634, η_*p*_^2^ = 0.00.

### 3.2. Neural results

Repeated ANOVA was performed with *Valence* (positive vs. negative vs. neutral) and *Time* (before mindfulness meditation or rest vs. after mindfulness meditation or rest) as the within-subject variable, *Group* (Mindfulness group vs. Non-mindfulness group) as the between-subject variable, fatigue as the covariable, and LPP amplitude as the dependent variable.

#### 3.2.1. LPP 300–600 (Pz, early window)

**FIGURE 3:**
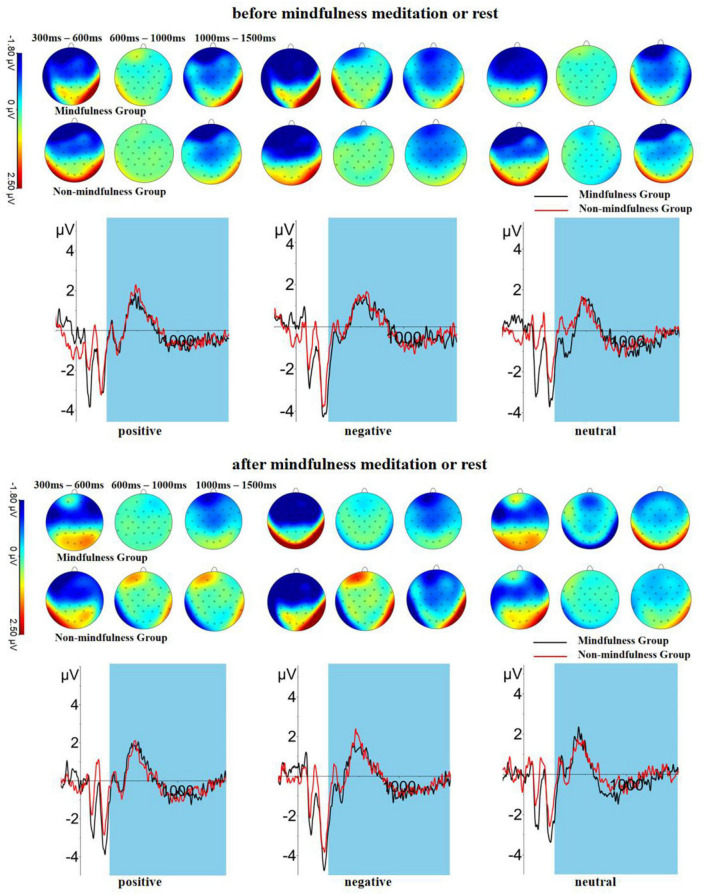
Late positive potential waveforms and scalp distributions in mean activity in response to different conditions between groups.

The results of the repeated measures ANOVA on LPP 300–600 (Pz, early window) are shown in Table 2 and Figure 3. The primary effect of *Valence* was significant, *F*(2,286) = 3.22, *p* = 0.041, η_*p*_^2^ = 0.02. The LPP amplitude of the positive stimuli was higher than the negative stimuli (*p* < 0.001). The LPP amplitude of the neutral stimuli was higher than that of the positive stimuli (*p* = 0.028). The main effect of *Time* was not significant, *F*(1,141) = 1.73, *p* = 0.191, η_*p*_^2^ = 0.01. The main effect of *Fatigue* was not significant, *F*(1,141) = 1.42, *p* = 0.235, η_*p*_^2^ = 0.01. The main effect of *Group* was significant, *F*(1,141) = 4.40, *p* = 0.038, η_*p*_^2^ = 0.03. However, after controlling the level of fatigue, the pairwise comparison of the group was not significant (*p*s > 0.05).

**TABLE 2:** Results of the repeated measures ANOVA on LPP 300–600 (Pz, early window).

Component	Factor	*df*	*F*	*p*	Partial η^2^
LPP 300–600 ms	Time	(1, 141)	1.73	0.191	0.01
	Valence	(2, 282)	3.22	0.041*	0.02
	Group	(1, 141)	4.40	0.038*	0.03
	Fatigue	(1, 141)	1.42	0.235	0.01
	Time × group	(1, 141)	0.16	0.687	0.00
	Time × fatigue	(1, 141)	0.63	0.428	0.00
	Valence × group	(2, 282)	0.78	0.459	0.01
	Valence × fatigue	(2, 282)	0.22	0.799	0.00
	Time × valence	(2, 282)	0.55	0.580	0.00
	Group × fatigue	(1, 141)	5.36	0.022*	0.04
	Time × group × fatigue	(1, 141)	0.03	0.871	0.00
	Valence × group × fatigue	(2, 282)	0.12	0.883	0.00
	Time × valence × group	(2, 282)	0.09	0.910	0.00
	Time × valence × fatigue	(2, 282)	0.71	0.493	0.01
	Time × valence × group × fatigue	(2, 282)	0.37	0.688	0.00

*p < 0.05.

**FIGURE 4:**
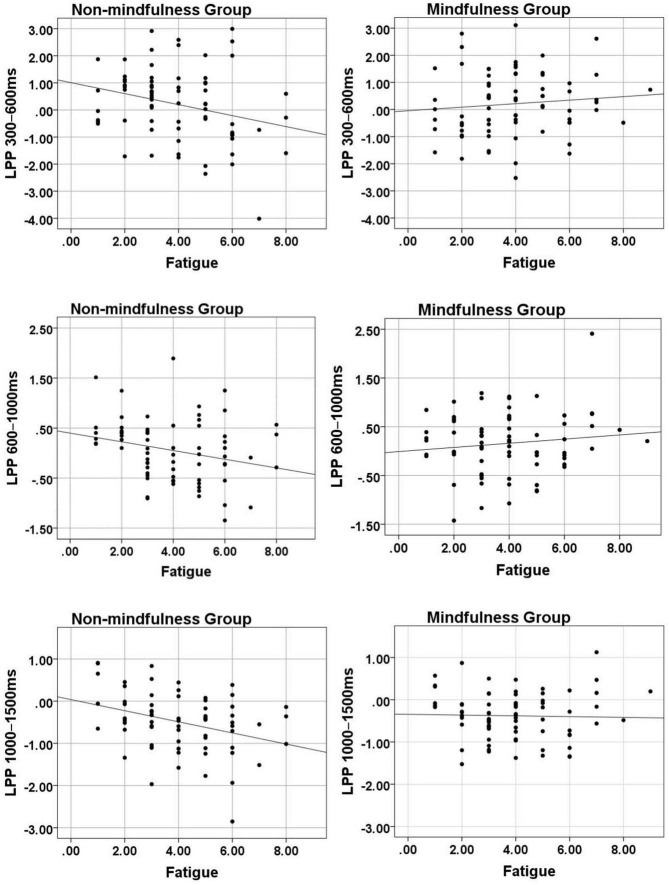
Correlations between the LPP and the level of fatigue in the mindfulness group and the Non-mindfulness group.

The interaction between *Group* and *Fatigue* was significant, *F*(1,141) = 5.36, *p* = 0.022, η_*p*_^2^ = 0.04. To further analyze the interaction between the *Group* and *Fatigue*, a correlations analysis was conducted between the amplitudes of LPP 300–600 and the levels of fatigue between groups (Figure 4). As shown in Table 3, there was a significant negative correlation between LPP 300–600 and fatigue levels in the Non-mindfulness group (*r* = −0.25, *p* = 0.037). However, there was no significant correlation between LPP 300–600 and fatigue levels in the Mindfulness group (*r* = 0.10, *p* = 0.397).

**TABLE 3:** Correlations between LPPs and the levels of fatigue between groups.

		*r*	*p*
LPP 300–600 ms	Mindfulness group	0.10	0.394
	Non-mindfulness group	−0.25	0.037*
LPP 600–1,000 ms	Mindfulness group	0.12	0.302
	Non-mindfulness group	−0.31	0.010*
LPP 1,000–1,500 ms	Mindfulness group	−0.03	0.808
	Non-mindfulness group	−0.34	0.004**

*p < 0.05, **p < 0.01.

There was no other significant interaction in the results of the repeated measures ANOVA on LPP 300–600 (all *p*s > 0.05).

#### 3.2.2. LPP 600–1,000 (Pz, middle window)

The results of the repeated measures ANOVA on LPP 600–1,000 (Pz, middle window) are shown in Tables 4, 5 and Figure 3. The main effect of *Valence* was significant, *F*(2,286) = 6.49, *p* = 0.002, η*p*^2^ = 0.04. The LPP amplitude of the negative stimuli was higher than the positive (*p* = 0.010) and neutral stimuli (*p* < 0.001). The LPP amplitude of the positive stimuli was higher than neutral stimuli (*p* < 0.001). The primary effect of *Time* was not significant, *F*(1,141) = 0.20, *p* = 0.657, η_*p*_^2^ = 0.00. The main effect of *Fatigue* was not significant, *F*(1,141) = 0.60, *p* = 0.439, η_*p*_^2^ = 0.00. The primary effect of *Group* was not significant, *F*(1,141) = 2.69, *p* = 0.103, η_*p*_^2^ = 0.02.

**TABLE 4:** Late positive potential amplitude (Pz) between the Mindfulness and Non-mindfulness groups under different valences.

Electrode	Time	Non-mindfulness group (*M* ± *SD*)			Mindfulness group (*M* ± *SD*)		
		**Positive**	**Negative**	**Neutral**	**Positive**	**Negative**	**Neutral**
LPP 300–600 ms	Before mindfulness meditation or rest	0.17 ± 1.54	−0.11 ± 1.64	0.17 ± 1.48	0.23 ± 1.42	−0.19 ± 1.37	−0.05 ± 1.38
	After mindfulness meditation or rest	0.40 ± 1.67	0.29 ± 1.70	0.27 ± 1.30	0.64 ± 1.46	0.09 ± 1.35	0.54 ± 1.43
LPP 600–1,000 ms	Before mindfulness meditation or rest	0.18 ± 0.81	0.23 ± 1.03	−0.23 ± 0.86	0.21 ± 0.86	0.41 ± 1.08	0.14 ± 0.93
	After mindfulness meditation or rest	0.00 ± 0.80	0.29 ± 0.90	−0.16 ± 0.98	0.17 ± 0.74	0.29 ± 0.95	−0.28 ± 0.78
LPP 1,000–1,500 ms	Before mindfulness meditation or rest	−0.54 ± 0.85	−0.64 ± 0.90	−0.43 ± 0.94	−0.34 ± 0.83	−0.50 ± 0.86	−0.33 ± 0.79
	After mindfulness meditation or rest	−0.53 ± 0.98	−0.55 ± 0.76	−0.24 ± 0.84	−0.40 ± 0.67	−0.51 ± 0.74	−0.20 ± 0.68

**TABLE 5:** Results of the repeated measures ANOVA on LPP 600–1,000 (Pz, middle window).

Component	Factor	*df*	*F*	*p*	Partial η^2^
LPP 600–1,000 ms	Time	(1, 141)	0.20	0.657	0.00
	Valence	(2, 282)	6.49	0.002**	0.04
	Group	(1, 141)	2.69	0.103	0.02
	Fatigue	(1, 141)	0.60	0.439	0.00
	Time × group	(1, 141)	0.25	0.621	0.00
	Time × fatigue	(1, 141)	1.71	0.194	0.01
	Valence × group	(2, 282)	0.46	0.630	0.00
	Valence × fatigue	(2, 282)	0.32	0.723	0.00
	Time × valence	(2, 282)	1.80	0.167	0.01
	Group × fatigue	(1, 141)	5.11	0.025*	0.03
	Time × group × fatigue	(1, 141)	1.54	0.217	0.01
	Valence × group × fatigue	(2, 282)	0.63	0.534	0.00
	Time × valence × group	(2, 282)	1.15	0.317	0.01
	Time × valence × fatigue	(2, 282)	1.11	0.332	0.01
	Time × valence × group × fatigue	(2, 282)	0.50	0.607	0.00

*p < 0.05, **p < 0.01.

The interaction between *Group* and *Fatigue* was significant, *F*(1,141) = 5.11, *p* = 0.025, η*p*^2^ = 0.03. To further analyze the interaction between the *Group* and *Fatigue*, a correlation analysis was conducted between the amplitudes of LPP 600–1,000 and the levels of fatigue between groups (shown in Figure 4). As shown in Table 3, there was a significant negative correlation between LPP 600–1,000 and fatigue levels in the Non-mindfulness group (*r* = −0.31, *p* = 0.010). However, there was no significant correlation between LPP 600–1,000 and fatigue levels in the Mindfulness group (*r* = 0.12, *p* = 0.397).

There was no other significant interaction in the results of the repeated measures ANOVA on LPP 600–1,000 (all *p*s > 0.05).

#### 3.2.3. LPP 1,000–1,500 (Pz, late window)

The results of the repeated measures ANOVA on LPP 1,000–1,500 (Pz, late window) are shown in Table 6 and Figure 3. The main effect of *Valence* was significant, *F*(2,286) = 4.35, *p* = 0.014, η_*p*_^2^ = 0.03. The LPP amplitude of the neutral stimuli was higher than the positive stimuli (*p* = 0.001), and the negative stimuli (*p* < 0.001). The main effect of *Fatigue* was significant, *F*(1,141) = 6.09, *p* = 0.015, η_*p*_^2^ = 0.04. The primary effect of *Time* was not significant, *F*(1,141) = 0.09, *p* = 0.771, η_*p*_^2^ = 0.00. The main effect of *Group* was not significant, *F*(1,141) = 2.40, *p* = 0.123, η_*p*_^2^ = 0.02.

**TABLE 6:** Results of the repeated measures ANOVA on LPP 1,000–1,500 (Pz, late window).

Component	Factor	*df*	*F*	*p*	Partial η^2^
LPP 1,000–1,500 ms	Time	(1, 141)	0.09	0.771	0.00
	Valence	(2, 282)	4.35	0.014*	0.03
	Group	(1, 141)	2.4	0.123	0.02
	Fatigue	(1, 141)	6.09	0.015*	0.04
	Time × group	(1, 141)	0.02	0.896	0.00
	Time × fatigue	(1, 141)	0.05	0.818	0.00
	Valence × group	(2, 282)	0.41	0.663	0.00
	Valence × fatigue	(2, 282)	0.39	0.677	0.00
	Time × valence	(2, 282)	0.19	0.824	0.00
	Group × fatigue	(1, 141)	4.64	0.033*	0.03
	Time × group × fatigue	(1, 141)	0.24	0.628	0.00
	Valence × group × fatigue	(2, 282)	0.22	0.801	0.00
	Time × valence × group	(2, 282)	1.41	0.245	0.01
	Time × valence × fatigue	(2, 282)	0.22	0.806	0.00
	Time × valence × group × fatigue	(2, 282)	1.96	0.143	0.01

*p < 0.05.

The interaction between *Group* and *Fatigue* was significant, *F*(1,141) = 4.64, *p* = 0.033, η_*p*_^2^ = 0.03. To further analyze the interaction between the *Group* and *Fatigue*, a correlations analysis was conducted between the amplitudes of LPP 1,000–1,500 and fatigue levels between groups (Figure 4).

As shown in Table 3, there was a significant negative correlation between LPP 1,000–1,500 and fatigue levels in the Non-mindfulness group (*r* = −0.34, *p* = 0.004). However, there was no significant correlation between LPP 1,000–1,500 and the levels of fatigue in the Mindfulness group (*r* = −0.03, *p* = 0.808).

There was no other significant interaction in the results of the repeated measures ANOVA on LPP 1000–1500 (all *p*s > 0.05).

## 4. Discussion

Although previous research has demonstrated that mindfulness could improve the individual processing of emotion, it remains unclear whether mindfulness can improve the emotional responses of fatigued adults. This study aimed to understand whether mindfulness meditation influences the associations between fatigue and emotional response and to understand the role of mindfulness. It is the first study to combine subjective measures, ERP techniques, and intervention techniques to examine whether mindfulness meditation reduces the negative association between fatigue and emotion during emotional processing (hypothesis of the study). In this section, we discuss the main findings of the present experiment and its implications.

Mindfulness was demonstrated to be beneficial to both fatigue and emotions. Individuals aware of mindfulness can respond to emotional situations in a non-judgmental, relaxed manner compared to individuals who have rapid, automatic, habitual responses to emotions. By enabling individuals to shift their way of thinking and focus on feeling in the present moment to reduce the impacts of emotions that individuals perceive, mindfulness can avoid the tendency to process and react to emotions too quickly and facilitate the development of a more adaptive observational response stance.

The present experiment provided psychophysiological evidence for the impact of mindfulness meditation on the association between fatigue and emotions. We found a significant main effect of mindfulness on LPP 300–600. However, after controlling for the level of fatigue, the pairwise comparison of the group was no longer significant. This finding showed that fatigue also influenced emotion, as suggested by previous research suggested (e.g., Zeidan et al., 2010; Grillon et al., 2015; Thomas, 2019; Kudesia et al., 2022). However, after including the impact of mindfulness, the possible negative emotional consequence along with mental fatigue was eliminated. This result indicated the negative relationship between fatigue and emotions and highlighted the positive influence of mindfulness on them.

Also, the findings of the present study showed that fatigue was negatively correlated with LPP amplitude in each phase during emotional processing, only in the Non-mindfulness group. This indicated that without mindfulness meditation, the more fatigued an individual is, the lower LPP amplitude they have when responding to emotional stimuli. In that case, what happens if the fatigued individual takes part in mindfulness meditation? In the Mindfulness group, the influence of fatigue on LPPs was minimized, with no significant association. Previous studies demonstrated that mindfulness promotes emotional stability (Shapiro et al., 2006; Pavlov et al., 2015; Schoenberg, 2016; Kaunhoven and Dorjee, 2017). Mindfulness meditation might also help individuals reduce the negative relationship between fatigue and emotions during emotional processing by being fully aware of the present fatigue state through open awareness and adjusting their awareness of emotional stimuli to better respond after emotional arousal.

*Thus, the hypothesis of the study is supported, suggesting that mindfulness would reduce the negative association between fatigue and emotional responses at the neural level.*

As mentioned above, being fatigued had a significant negative association with individual emotional experience and emotional processing (e.g., increase in negative emotions, decrease in attention and positive emotions, and reduced performance during the task) (Maslach et al., 2001; Alarcon et al., 2009; Li et al., 2016). The non-significant correlation between fatigue and the amplitudes of LPP was found in all of the early and late time windows during emotional processing among the Mindfulness group. This result was consistent with the existing literature that mindfulness impacts both the bottom-up and top-down processes of emotions (Chen and Deng, 2022). Models of the brain systems involved in emotion regulation summarized two processes underlining the neural bases of the influence of mindfulness on emotions: a bottom-up process that functions on the affective properties of stimuli and a higher-level cognitive process that acts on the individual difference in emotion and regulatory abilities (Deng et al., 2019). For example, individuals with high mindfulness levels are less affected by undesirable emotional stimuli in the process of emotional regulation in the early stage of emotional processing; and can use fewer cognitive resources to regulate negative stimuli in the later stage of emotional processing (Deng et al., 2021). In this case, mindfulness might buffer the negative emotional consequences along with the mental fatigue in both the bottom-up and top-down processing of emotion.

For the bottom-up processes, attention networks are considered to be more involved during mindfulness meditation since it helps people regulate and reorient their attention to the present moment (Reva et al., 2014). For example, an ERP study examining the influence of mindfulness meditation on brain activations during emotional processing (Deng et al., 2019) showed that P1 amplitudes decreased after mindfulness, which might reflect the impact of mindfulness on the bottom-up processes during emotional processing. Similarly, in the present study, mindfulness meditation might facilitate the bottom-up attentional process of the high-fatigue participants by reorienting their attention from the fatigued experience to the present moment; and reducing the negative relationship between fatigue and emotion on emotional responses at the neural level.

For the top-down processes, mindfulness benefits individuals’ present experience, and might directly promote higher-level processing of emotions. For example, a previous study found that mindfulness practices reduced the requirements of cognitive resources in the late stage of emotional processing in early adolescents (Deng et al., 2019). Moreover, a previous study examined differences in frontal EEG asymmetry during emotion regulation between participants who had different levels of trait mindfulness (Deng et al., 2021). As a biomarker for effective emotion regulation and regulatory ability, greater left (relative to right) frontal alpha band asymmetry was found among high-mindfulness adolescents than among low-mindfulness adolescents during emotion regulation. The same might happen in the present study, in which mindfulness might promote the effectiveness of emotion regulation of the high-fatigue participants during emotional processing. Mindfulness helps them to improve their regulatory abilities and mental resilience to fatigued experiences and reduce the negative association between fatigue and emotional responses at the neural level.

Surprisingly, the present study found clear evidence of fatigue affecting the late-phase LPP amplitudes which rarely showed in previous research. The findings showed that fatigue significantly affects the neural responses to emotional stimuli in the late stage of emotional processing. One potential mechanism is that fatigue reflects a status of scarce cognitive resources. The change of LPP amplitude in the late window reflects input from cognitive resources such as attention (Hajcak et al., 2010) and emotional stimuli (Cuthbert et al., 2000; Hajcak and Olvet, 2008). During emotion regulation, individuals use cognitive resources to respond and adjust their processing and experience of emotions, regardless of their response to the emotional stimulus. Therefore, with limited cognitive resources, fatigued individuals would have a lower late phase of the LPP (1,000–1,500 ms).

The negative correlation between fatigue and LPP (1,000–1,500 ms) observed in the experiment prompted that fatigue impairs emotion processing and emotion regulation. In the workplace, high emotional demands and long work hours cause fatigue and consume many emotional resources. Employees who require high emotional resources do not simply respond to clients’ emotions at work but are driven by their work’s emotional and motivational meaning and engage in deeper emotional processing and emotion regulation by mobilizing their cognitive resources to achieve emotional empathy with clients. When employees are fatigued, they experience significant mental exhaustion and a reduced ability to respond to multiple stimuli (Sadeghniiat-Haghighi and Yazdi, 2015). They may not have the added resources to respond to further emotional stimuli. In this case, we suggested that individuals improve their processing of emotions in a fatigued state through mindfulness meditation training rather than simply resting.

The differences in the LPP amplitudes for different valences reflect the degree of neural responses, selected attention, and cognitive resource involvement to the emotional stimuli among the participants (Hajcak and Olvet, 2008). The amplitude of LPP is generally but not always positive. In the present study, the means of the amplitudes of the early and mid LPPs were positive values. However, the mean of the amplitude of the late LPP was negative. The result might reflect a decrease in the attention and regulation of the emotional stimuli in the later stage of emotional processing. The negative correlations between fatigue and the LPPs in different time windows showed a consistent relation between participants’ fatigue level and their responses to different emotions. Specifically, the higher fatigue participants experienced, the lower level of neural responses, attention, and cognitive resource involvement to the emotional stimuli they presented.

### 4.1. Limitations and future directions

A few limitations should be discussed before generalizing this study’s results. In this study, fatigue was measured using a single-item subjective report. Although such a measurement was validated and reliable in previous studies, it would be more interesting to set up a series of tasks (e.g., a 40-minute 2-back task) to induce mental fatigue. In addition, this study used emotional images as stimuli to elicit emotional responses. Although such materials effectively evoke basic social emotions, complex emotional scenarios will be more conducive to exploring emotional responses in real social situations. Thus, future research could set up a more complex emotional scenario or specific event related to real-life situations to elicit emotional responses.

In the present study, participants were randomly assigned to either the Mindfulness or the Non-mindfulness groups. Participants in the Mindfulness group completed a 15-minute mindfulness meditation, which involved a breath-based mindfulness exercise and a body exploration exercise. The duration of the mindfulness meditation was relatively short. Different from the neural results, we didn’t find any significant interaction between group, valence and time in behavioral rating. It is possible that the neural response might be more sensitive to the effect of short time mindfulness meditation than the self-ratings of emotional experience. The change of the self-report emotional experience is on a conscious level and might be harder to be changed in a short time scale through mindfulness meditation. Therefore, in the future study, it is important for researchers to examine the effect of mindfulness meditation on a longer time scale and by using different mindfulness practices.

The present study on mindfulness and emotional processing mainly focused on participants’ fatigue levels. However, individual differences (e.g., mental health status and personality) could potentially influence on the practice of mindfulness and the processing of different emotions. Future studies should include more relevant variables to increase the generalizability of the results.

This study found evidence of mindfulness effectively mitigating the negative association between fatigue and emotional responses, and the clear negative correlation between fatigue and the LPP late windows. The next question is how fatigue impairs higher-level top-down emotional processes. Future studies should use more sophisticated designs and analyses to examine the specific processes between mindfulness, fatigue, and emotion, to further understand its mechanism. For example, whether this is achieved by influencing personal calls on attentional resources.

## 5. Conclusion

The study findings showed a clear negative correlation between fatigue and LPP late windows. Importantly, we found that mindfulness meditation, to some extent, offsets the negative association between fatigue and the neural activation of emotion, suggesting that mindfulness can be used as a solution in scenarios where individuals will feel fatigued, such as in the workplace. Mindfulness can help individuals who have emotional demands in their studies or at work to improve their processing of emotions in a fatigued state.

## Data availability statement

The raw data supporting the conclusions of this article will be made available by the authors, without undue reservation.

## Ethics statement

The studies involving human participants were reviewed and approved by the Shenzhen University Ethics Review Committee. The patients/participants provided their written informed consent to participate in this study.

## Author contributions

JF and XD proposed the research questions, designed the study, and contributed to the key revisions of the manuscript. ML and XL contributed to the data collection, data analysis, and data interpretation. JF and WL drafted the manuscript. All authors approved the final version of the manuscript for submission.
