# Supplementary material for: Effects of mindfulness and fatigue on emotional processing: an event-related potentials study
Source: Front Behav Neurosci. 2023 May 25;17:1175067. doi: 10.3389/fnbeh.2023.1175067 (PMC10249016; doi:10.3389/fnbeh.2023.1175067)
Supplement: Supplementary file 1 [file Presentation_1.pdf]

Supplementary 1

Audio guidance of breath-focused mindfulness meditation in Chinese: 这个练习是呼吸觉察，你可以坐着、躺着或站着练习，我们大概会练习十分钟，在这个练习中，我们将感受生命中最重要的元素—呼吸。我们将感受自己呼吸的感觉，和呼吸在一起，全然地感受呼吸……让我们静静地感觉此刻，坐着，躺着或站着，感觉当下身体在这个姿势的感受……接着，让我们把注意力轻轻地放到我们的呼吸，感受自己正在呼吸，也许呼吸的时候明显的身体感觉发生在我们的鼻端，感受气息自然的进出我们的鼻端。或者我们会感受在我们呼吸的时候，我们的腹部会随着我们的呼吸缓缓的上升，下降。无论是鼻端或是腹部，你可以选择其中一个身体的感受，让我们的心就放在我们的呼吸，轻轻地跟着，自然地呼吸，不用刻意的放慢，也不用刻意的加快我们的呼吸，就让身体自己呼吸，我们只是静静的感受它……觉知我们的呼吸，没有任何的目的，没有为了达到什么，也没有什么去除掉什么，只是单纯的感受自己还有呼吸，还在呼吸……单纯的让我们的心随着呼吸的律动，跟着它轻轻地，知道自己正在呼吸的感觉……往往当我们注意呼吸时，心不会乖乖地停留在呼吸上，心似乎有它自己的生命，它会离开呼吸，跑到过去，跑到未来。没有关系，每个人都会如此，当我们发现心不在呼吸上，我们只需要知道我们的心离开了呼吸，然后轻轻地把我们的注意力再带回到我们的呼吸，再次的去感受呼吸的时候身体最明显的部位，或许是鼻端，或许是腹部……一遍又一遍，当我们的心离开了呼吸，轻轻地将我们的心带回来，回到呼吸……如果可以，试着让我们的心，轻轻地跟着每一个吸气，每一个呼气，随着呼吸的律动，缓缓地跟着……没有任何的目的，没有为了什么，只是单纯的和自己的呼吸在一

起，单纯的感受自己，还有呼吸……这个练习即将结束，让我们感谢自己愿意抽出时间来练习照顾好自己。最后，让我们祝福自己，愿我幸福快乐，愿我幸福快乐，愿我幸福快乐！

The English translation for the breath-focused mindfulness meditation: This practice is to feel our breath; we can stand, lie down or sit. We will spend ten minutes on this practice. During the practice, we will experience the most important element of our lives- the breath. We will experience our breathing, be with our breathing, and fully feel our breathing....... Let's enjoy the present moment quietly sitting, lying, or standing. Just enjoy the sense that comes from the posture. Let’s turn our attention to our breath, and feel that we are breathing. Perhaps the most obvious body sensation occurs at our nose, the breath naturally flows in and out of the nose. Also, when we are breathing, the belly goes up and down with each breath. We can choose to focus on the nose or the belly, and put our heart into the breath, following it softly. Breathing naturally, we don't need to try to slow down or accelerate our breathing, we can just let our body breathe by itself, and we can just feel it quietly. Let’s be aware of our breath, without the intention of either achieving, or ignoring anything, just feel we are breathing, and let our heartbeat follow the rhythm of our breath, following it lightly. Learn the sensation of breathing. Sometimes, when we focus on our breath, the mind will not stay there obediently, it seems that our mind has its own life, it will leave the breath, run back to the past, and run to the future. But take it easy, everyone experiences this. When

we find out that our mind is not on our breath, we only need to know that it leaves, and pay attention to our breath again, feeling the most visible part of our body when we are breathing; the nose or the belly, again and again. When our mind departs from our breath, take it back lightly, back to the breath. If it's all right, try to let your heart, gently follow each inhalation, each exhalation, with the rhythm of the breath, softly. Follow, without any other goal than to be just with your breath, merely feel yourself, and breathe. The training is at the end; let's thank ourselves for taking the time to practice taking care of ourselves. Lastly, let's bless ourselves. Wish me happy! Wish me happy! Wish me happy!

Audio guidance of body scanning mindfulness meditation in Chinese:我们即将要开始一段关于正念身体扫描的练习。你只需要在这个过程中全然去感受，不需要对我做出任何回应。现在，我邀请你把你的眼睛闭上，把你的双脚轻轻地放在地板上，感受你的双脚和地面接触的感觉，去细细地体会你的双脚和地面接触的那个部分，给你带来怎样的感觉。接下来，让我们的感觉稍微向上移动一点点，让我们来感觉我们的双脚与我们的鞋子接触的感觉，感觉你的双脚在鞋子的包裹当中，有一些怎样的感觉，是紧的，还是松的，是暖暖的，还是有一点凉凉的。接下来，让我们来感觉一下我们的小腿，感觉一下我们的小腿此时此刻的感觉。我们的小腿在这个当下，有一些什么样的感觉呢？是紧张的，还是放松的？让我们关注这份感觉，并且把我们的感觉停留在那待一会。接下来，让我们的感觉往上移动一点，来感觉一下我们的大腿，感觉我们的大腿跟椅子接触的那个部分，感受一下

是有一些紧紧的，硬硬的，还是软软的，让我们把感觉停留在那个部分，去感觉我们的大腿跟椅子接触的那个感觉。接下来，让我的感觉继续往上移动，感觉我们的双手，去感觉我们的双手正在与哪个部分接触，是我们的身体还是某一个物件，让我们来感受我们的双手和这个部分接触的感觉，所有的感觉我们都可以去感受，所有的感觉我们都可以去接纳。接下来，让我们的感觉继续往上移动，让我们来感受我们的头部和那顶帽子接触的部分带给你怎样的感觉，紧紧的还是松松的？凉凉的还是暖暖的？让我们来感受我们的头部此时此刻的感觉，就把我们的感觉停留在那。接下来，让我们来感觉一下我们的呼吸，感受气体从外部流向我们的鼻腔，再流进我们的身体，在从我们的身体流进我们的鼻腔，最后与我们的嘴唇触碰，再流出的那个感觉，将我们的注意力完全停留在我们的鼻端，去感受我们的呼吸。这个练习即将结束，现在，我邀请你对自己说：我关注我的每一个感受，我关注我的每一个当下，我关注每一个此时此刻，我愿意去关注、接纳所有发生在我面前的一切。

The English translation for the body scanning mindfulness meditation: Let's do a mindfulness exercise of body scanning. You just need to be engaged in the process of feeling, do not need to respond to me. Now, I invite you to close your eyes; and place your feet gently on the floor. Notice how your feet feel when they touch the ground. Next, let's move our senses up a little bit. Let's feel how our feet touch our shoes. Let's feel how our feet feel inside the shoes. Is it tight or loose, warm or cold? Next, let's focus our senses on our calves. Feel what our calves feel like right now. What do our

calves feel like at this moment? Nervous or relaxed? Let's focus on this feeling and let our feeling stay there for a while. Now, let's move our senses up a little bit to the thighs. Feel part of our thighs touching the chair. Is it tight, hard, or soft? Let our senses stay there. Feel how our thighs touch the chair. Next, let our senses continue to move up to feel our hands. What is our hand touching? Is it a part of our body or an object? To feel how our hands feel right now. All the feelings we can accept. All feelings are acceptable to us. Now, let's move our senses up again. Feel how our head feels when it touches the hat. Is it loose or tight? Is it cool or warm? Let's feel what our heads are feeling right now, and just leave our feelings stay there. Next, let's focus on our breathing. Feel the air coming in from the outside into our nose, then into our body, then out of our body into our nose, touching our lips, and then out again. Bring our attention to the nose, feel our breath. This exercise is coming to an end. I invite you to say to yourself: I am aware of all my feelings. I pay attention to each moment. I am willing to accept and pay attention to everything that happens to me.
